# Supplementary material for: Maternal hypertensive disorders in pregnancy and early childhood cardiometabolic risk factors: The Generation R Study
Source: PLoS One. 2021 Dec 23;16(12):e0261351. doi: 10.1371/journal.pone.0261351 (PMC8699579; doi:10.1371/journal.pone.0261351)
Supplement: S1 File — (DOCX) [file pone.0261351.s001.docx]

# Supplementary material.

**Maternal hypertensive disorders in pregnancy and early childhood cardiometabolic risk factors: the Generation R Study.**

D.V. Gootjes^1,2^, A.G. Posthumus^1,2^, V.W.V Jaddoe^2,3^, B.B. van Rijn^1,2^, E.A.P. Steegers^1,2^

^1^Department of Obstetrics and Gynaecology, Division of Obstetrics and Fetal Medicine, ^2^Generation R Study Group. ^3^Department of Paediatrics, all: Erasmus University Medical Centre, Rotterdam, The Netherlands.

**
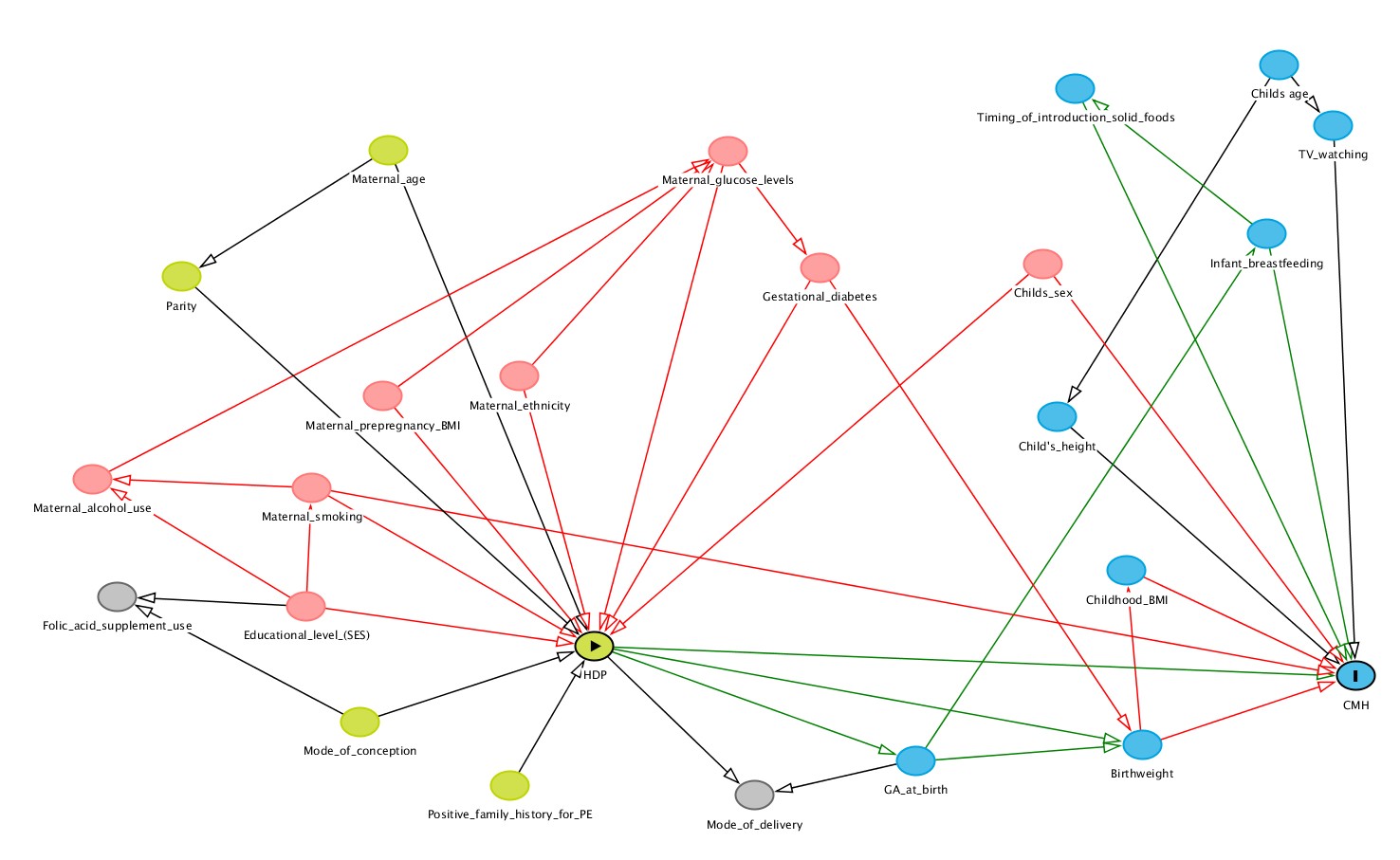
S1 Figure. Directed Acyclic Graph (DAG) representing the pathways between maternal HDP and childhood cardiometabolic risk factors.**

Abbreviations: HDP, hypertensive disorders of pregnancy; GA, gestational age; BMI, body mass index; CMH, cardiometabolic health; SES, socioeconomic status; PE, pre-eclampsia; TV, television. The confounders (red) were included in the confounder regression models.

**S1 Table. Offspring parameters according to the certain HDP that the mother experienced.**

| **Study population**  **n = 7794** | **Gestational hypertension (n=293)** | **PE**  **(n=139)** | **HELLP**  **(n=14)** | **PE and HELLP**  **(n=20)** | **Superponated PE/HELLP (n=25)** |
| --- | --- | --- | --- | --- | --- |
| **Child characteristics** |  |  |  |  |  |
| Male sex, n (%) | 139 (47.4%) | 68 (48.9%) | 8 (57.1%) | 11 (55.0%) | 13 (52.0%) |
| Gestational age at birth  (weeks) | 40.1 (37.0-42.1) | 40.0 (37.0-41.9) | 40.1 (38.0-42.2) | 40.5 (37.8-41.4) | 40.4 (36.1-41.9) |
| Birth weight (grams) | 3407 (528) | 3353 (553) | 3616 (504) | 3360 (380) | 3493 (522) |

Abbreviations: BMI, body mass index; HDP, hypertensive disorder of pregnancy. Values are percentages for categorical variables, means (SD) for continuous variables with a normal distribution, or medians (5^th^, 95^th^ percentile) for continuous variables with a skewed distribution.

**S2 Table.** **Baseline characteristics of the population in- and excluded from analyses.**

|  | **Total prenatal cohort**  **N = 8976** | **Study population**  **n = 7794** | **Excluded**  **n = 1182** | **p-value** |
| --- | --- | --- | --- | --- |
| **Maternal characteristics** |  |  |  |  |
| Age at intake (years) | 30.3 (5.3-37.9) | **30.2 (20.2-37.9)** | **30.6 (21.0-38.1)** | **0.02** |
| Pre-pregnancy BMI (kg/m^2^) | 22.8 (18.7-31.6) | 22.7 (18.6-32.4) | 22.8 (18.7-31.6) | 0.75 |
| High educational level, n (%) | 3584 (39.9%) | 3073 (39.4%) | 511 (43.2%) | 0.08 |
| Dutch and Western ethnicity, n (%) | 5194 (57.9%) | 4479 (57.5%) | 715 (60.5%) | 0.20 |
| Nulliparous, n (%) | 4905 (54.6%) | 4260 (55.4%) | 645 (54.6%) | 0.89 |
| Never smoked in pregnancy, n (%) | 6509 (72.5%) | 5651 (72.5%) | 859 (72.7%) | 0.12 |
| Never alcohol in pregnancy, n (%) | 4544 (50.6%) | 3940 (50.6%) | 604 (51.1%) | **<0.001** |
| **Child characteristics** |  |  |  |  |
| Male sex, n (%) | 4531 (50.05%) | 3952 (50.7%) | 579 (49.0%) | 0.29 |
| Gestational age at birth, (weeks) | 40.1 (36.4-42.0) | 40.1 (36.9-42.1) | 39.7 (35.3-41.9) | **<0.001** |
| Birth weight (g) | 3390 (583) | 3415 (561) | 3198 (687) | **<0.001** |
| Ever breast feeding, n (%) | 5667 (63.1%) | 4950 (63.5%) | 717 (60.7%) | 0.08 |
| Breastfeeding duration (months) | 4.8 (3.8) | 4.8 (3.8) | 4.7 (3.7) | 0.45 |

Abbreviations: BMI, body mass index; HDP, hypertensive disorder of pregnancy. Values are percentages for categorical variables, means (SD) for continuous variables with a normal distribution, or medians (5^th^, 95^th^ percentile) for continuous variables with a skewed distribution. Confounders are imputed. Non-imputed values are presented as valid percentages. Differences in baseline characteristics were tested using Students t-test, Mann-Whitney and chi-square tests.

**S3 Table: Sensitivity analyses of hypertensive disorders of pregnancy (cases defined only by PE and HELLP) and childhood cardiometabolic risk factors.**

|  | **Cardiometabolic risk factor** |  | **Model** |  |  |  |
| --- | --- | --- | --- | --- | --- | --- |
|  |  |  | **Basic** |  | **Confounder** |  |
|  |  | **n** | **β (95% CI)** | **p-value** | **β (95% CI)** | **p-value** |
|  |  |  |  |  |  |  |
| **2 months** | **BMI** | **3779** | -0.14 (-0.34 ; 0.06) | 0.16 | -0.12 (-0.32 ; 0.07) | 0.22 |
| **6 months** | **BMI** | **4518** | **-0.19 (-0.38 ; -0.002)** | **0.048** | -0.18 (-0.37 ; 0.01) | 0.06 |
| **12 months** | **BMI** | **4614** | **-0.20 (-0.38 ; -0.01)** | **0.04** | **-0.19 (-0.37 ; -0.01)** | **0.04** |
| **36 months** | **BMI** | **3933** | -0.09 (-0.29 ; 0.11) | 0.36 | -0.07 (-0.26 ; 0.13) | 0.52 |
| **6 years** | **Systolic blood pressure** | **4874** | 0.10 (-0.08 ; 0.27) | 0.29 | 0.08 (-0.10 ; 0.25) | 0.39 |
|  | **Diastolic blood pressure** | **4874** | 0.09 (-0.09 ; 0.27) | 0.31 | 0.07 (-0.11 ; 0.24) | 0.45 |
|  | **BMI**¶ | **5312** | 0.05 (-0.12 ; 0.22) | 0.59 | 0.03 (-0.13 ; 0.19) | 0.70 |
|  | **Fat mass index** ¶ | **5163** | 0.08 (-0.09 ; 0.24) | 0.34 | 0.05 (-0.10 ; 0.21) | 0.51 |
|  | **Cholesterol**¶ | **3531** | -0.03 (-0.23 ; 0.18) | 0.78 | -0.03 (-0.24 ; 0.17) | 0.76 |
|  | **Triglycerides**¶ | **3523** | -0.06 (-0.27 ; 0.15) | 0.58 | -0.06 (-0.27 ; 0.15) | 0.58 |
|  | **Cardiometabolic risk factor clustering** | **3196** | 1.15 (0.97 ; 1.35) | 0.41 | 1.10 (0.66 ; 1.83) | 0.71 |

Abbreviations: BMI, body mass index; HDP, hypertensive disorder of pregnancy. Values are regression coefficients (95% confidence interval) that reflect the difference in childhood outcomes in SD scores, in pregnancies complicated by HDP versus pregnancies not complicated by HDP. Basic model was adjusted for child’s sex. Confounder model includes maternal pre-pregnancy body mass index, educational level, ethnicity, smoking during pregnancy, alcohol use during pregnancy, maternal glucose levels and presence of gestational diabetes. ¶Variables were log transformed.

**S4 Table.** **Observed and expected values of confounders.**

|  | **Observed** | **Expected** |
| --- | --- | --- |
| **Maternal characteristics** |  |  |
| Pre-pregnancy BMI (kg/m^2^) | 22.7 (18.6-32.4) | 22.7 (18.6-32.4) |
| High educational level, n (%) | 3073 (39.4%) | 2934 (41.5%) |
| Dutch and Western ethnicity, n (%) | 4479 (57.5%) | 4310 (58.7%) |
| Never smoked in pregnancy, n (%) | 5651 (72.5%) | 4946 (727%) |
| Never alcohol in pregnancy, n (%) | 3940 (50.6%) | 3335 (49.7%) |
| Gestational diabetes, n (%) | 85 (1.1%) | 81 (1.1%) |
| Glucose (mmol/L) | 4.4 (0.8) | 4.4 (0.8) |
| **Child characteristics** |  |  |
| Male sex, n (%) | 3952 (50.7%) | 3928 (50.7%) |

Abbreviations: BMI, body mass index. Values are percentages for categorical variables, means (SD) for continuous variables with a normal distribution, or medians (5^th^, 95^th^ percentile) for continuous variables with a skewed distribution. Non-imputed values are presented as valid percentages. Differences in baseline characteristics were tested using Students t-test, Mann-Whitney and chi-square tests.
