# Supplementary material for: Maternal hypertensive disorders in pregnancy and early childhood cardiometabolic risk factors: The Generation R Study
Source: PLoS One. 2021 Dec 23;16(12):e0261351. doi: 10.1371/journal.pone.0261351 (PMC8699579; doi:10.1371/journal.pone.0261351)
Supplement: Attachment — Submitted filename: Rebuttal PONE-D-21-12261.docx [file pone.0261351.s002.docx]

**We thank the reviewers and the editorial office of PLOS ONE for the opportunity to submit major revisions. We appreciate the reviewers’ elaborate suggestions that have improved our manuscript. Our answers (in bold) are as follows**

Journal Requirements:

When submitting your revision, we need you to address these additional requirements.

1. Please ensure that your manuscript meets PLOS ONE's style requirements, including those for file naming. The PLOS ONE style templates can be found at <https://journals.plos.org/plosone/s/file?id=wjVg/PLOSOne_formatting_sample_main_body.pdf> and [https://journals.plos.org/plosone/s/file?id=ba62/PLOSOne_formatting_sample_title_authors_affiliations.pdf](about:blank)

**We apologize for the inconvenience and made adjustments according to the instructions, following the templates proved on the PLOS ONE website.**

2. Thank you for submitting the above manuscript to PLOS ONE. During our internal evaluation of the manuscript, we found significant text overlap between your submission and the following previously published works: file:///C:/Users/jvostmyer/Downloads/140625_Gaillard-Romy.pdf

We would like to make you aware that copying extracts from previous publications, especially outside the methods section, word-for-word is unacceptable. In addition, the reproduction of text from published reports has implications for the copyright that may apply to the publications.

Please revise the manuscript to rephrase the duplicated text, cite your sources, and provide details as to how the current manuscript advances on previous work. Please note that further consideration is dependent on the submission of a manuscript that addresses these concerns about the overlap in text with published work. We will carefully review your manuscript upon resubmission, so please ensure that your revision is thorough.

**Unfortunately, we are not able to open the link since it’s leading to a paper saved on the C-storage. Since dr. Gaillards’ thesis is about maternal hypertensive disorders within the Generation R population, little overlap on textual parts, especially the methods section, is possible. Obviously, we would like to address this point and make textual changes.**

3. For more information on PLOS ONE's expectations for statistical reporting, please see <https://journals.plos.org/plosone/s/submission-guidelines.#loc-statistical-reporting>. Please update your Methods and Results sections accordingly.

**The Methods and Results section was adapted according to the PLOS ONE’s guidelines.** **As suggested, the full results of any regression analysis performed is added as a supplementary file. This includes all estimated regression coefficients, their standard error, p-values, and confidence intervals, as well as the measures of goodness of fit.**

4. We note that you have indicated that data from this study are available upon request. PLOS only allows data to be available upon request if there are legal or ethical restrictions on sharing data publicly. For information on unacceptable data access restrictions, please see <http://journals.plos.org/plosone/s/data-availability#loc-unacceptable-data-access-restrictions>.

In your revised cover letter, please address the following prompts:

a) If there are ethical or legal restrictions on sharing a de-identified data set, please explain them in detail (e.g., data contain potentially identifying or sensitive patient information) and who has imposed them (e.g., an ethics committee). Please also provide contact information for a data access committee, ethics committee, or other institutional body to which data requests may be sent.

b) If there are no restrictions, please upload the minimal anonymized data set necessary to replicate your study findings as either Supporting Information files or to a stable, public repository and provide us with the relevant URLs, DOIs, or accession numbers. Please see <http://www.bmj.com/content/340/bmj.c181.long> for guidelines on how to de-identify and prepare clinical data for publication. For a list of acceptable repositories, please see <http://journals.plos.org/plosone/s/data-availability#loc-recommended-repositories>.

We will update your Data Availability statement on your behalf to reflect the information you provide.

**The datasets generated and analyzed during the current study are not publicly available due to individual privacy consideration. However, they are available from the data managers (Claudia J. Kruithof, c.kruithof@erasmusmc.nl or datamanagementgenr@erasmusmc.nl) and Director Generation R, Vincent Jaddoe (v.jaddoe@erasmusmc.nl) after a written agreement about the use of the data made via the Technology Transfer Office of Erasmus MC. We made adjustments accordingly in the revised cover letter.**

5. We note that you have included the phrase “data not shown” in your manuscript. Unfortunately, this does not meet our data sharing requirements. PLOS does not permit references to inaccessible data. We require that authors provide all relevant data within the paper, Supporting Information files, or in an acceptable, public repository. Please add a citation to support this phrase or upload the data that corresponds with these findings to a stable repository (such as Figshare or Dryad) and provide and URLs, DOIs, or accession numbers that may be used to access these data. Or, if the data are not a core part of the research being presented in your study, we ask that you remove the phrase that refers to these data.

**In the new, revised version of the manuscript, we added the data on pulse as outcome and the ponderal index as outcome measurement instead of BMI at 2, 6, 12 and 36 months (Table 2).**

6. Thank you for stating the following in the Acknowledgments Section of your manuscript:

'The Generation R Study was made possible by financial support from Erasmus MC, University
Medical Centre Rotterdam, the Netherlands; the Netherlands Organization for Health Research
and Development; the Netherlands Organization for Scientific Research; the Ministry of Health,
Welfare and Sport; and the Ministry of Youth and Families. Prof. Dr. Vincent Jaddoe received
additional grants from the Netherlands Organization for Health Research and Development
(grants 907.00303 and 916.10159, and VIDI 016.136.361) and a Consolidator Grant from the
European Research Council (ERC-2014-CoG-64916).

We note that you have provided funding information that is not currently declared in your Funding Statement. However, funding information should not appear in the Acknowledgments section or other areas of your manuscript. We will only publish funding information present in the Funding Statement section of the online submission form. Please remove any funding-related text from the manuscript and let us know how you would like to update your Funding Statement. Currently, your Funding Statement reads as follows: 'The author(s) received no specific funding for this work.'

Please include your amended statements within your cover letter; we will change the online submission form on your behalf.

**This statement is added in the cover letter:**

**Funding Statement: The Generation R Study is made possible by financial support from Erasmus Medical Center, Erasmus University Rotterdam, Rotterdam, and the Netherlands Organization for Health Research and Development (ZonMw). The funders had no role in the design of the study, the data collection and analyses, the interpretation of data, or the preparation of, review of, and decision to submit the manuscript.**

7. Please upload a copy of Figure 1, to which you refer in your text on page 27. If the figure is no longer to be included as part of the submission please remove all reference to it within the text.

**We apologize, this figure was uploaded separately since it was assumed this was according to the submitting guidelines. It is now included in the text again.**

8. Please include captions for your Supporting Information files at the end of your manuscript, and update any in-text citations to match accordingly. Please see our Supporting Information guidelines for more information: [http://journals.plos.org/plosone/s/supporting-information](about:blank).

**We now listed the supporting information with name and number, including a one-line title, at the end of your manuscript file.**

**Comments to the Author**
1. Is the manuscript technically sound, and do the data support the conclusions?
The manuscript must describe a technically sound piece of scientific research with data that supports the conclusions. Experiments must have been conducted rigorously, with appropriate controls, replication, and sample sizes. The conclusions must be drawn appropriately based on the data presented.

Reviewer #1: Partly

Reviewer #2: Yes

 2. Has the statistical analysis been performed appropriately and rigorously?

Reviewer #1: Yes

Reviewer #2: Yes

 3. Have the authors made all data underlying the findings in their manuscript fully available?
The [PLOS Data policy](https://eur01.safelinks.protection.outlook.com/?url=http%3A%2F%2Fwww.plosone.org%2Fstatic%2Fpolicies.action%23sharing&data=04%7C01%7Cd.gootjes%40erasmusmc.nl%7Ca75622a1803c4daada8108d93d1b218d%7C526638ba6af34b0fa532a1a511f4ac80%7C0%7C1%7C637608009022113790%7CUnknown%7CTWFpbGZsb3d8eyJWIjoiMC4wLjAwMDAiLCJQIjoiV2luMzIiLCJBTiI6Ik1haWwiLCJXVCI6Mn0%3D%7C1000&sdata=bw%2Fwkjd3RlTs6J%2Bn05mGSEm9aGLvgz7IWpOqDE0OcV4%3D&reserved=0) requires authors to make all data underlying the findings described in their manuscript fully available without restriction, with rare exception (please refer to the Data Availability Statement in the manuscript PDF file). The data should be provided as part of the manuscript or its supporting information, or deposited to a public repository. For example, in addition to summary statistics, the data points behind means, medians and variance measures should be available. If there are restrictions on publicly sharing data—e.g. participant privacy or use of data from a third party—those must be specified.

Reviewer #1: No

Reviewer #2: No

4. Is the manuscript presented in an intelligible fashion and written in standard English?
PLOS ONE does not copyedit accepted manuscripts, so the language in submitted articles must be clear, correct, and unambiguous. Any typographical or grammatical errors should be corrected at revision, so please note any specific errors here.

Reviewer #1: Yes

Reviewer #2: No

 5. Review Comments to the Author

Reviewer #1:

In the methods, it was described that maternal HDPs were defined at PE, GH, and HELLP. Do you have a data chart describing the average blood pressures, liver enzymes, etc. to provide evidence for these classifications?

**As suggested by the reviewer, systolic and diastolic blood pressure measured in the third trimester of pregnancy were added to the baseline characteristics (Table 1). Unfortunately, no data on liver enzymes was present, since the diagnosis was based on medical records. Therefore, blood samples derived at the research center, do not solely represent the blood samples at the time of diagnosis in hospital.**

Also, do all of the PE patients exhibit proteinuria? Some literature reports discuss PE to be defined as elevated blood pressure, with or without proteinuria. If the PE cases don't have proteinuria, what is the distinguishing factor of the PE cases from the GH cases?

**Yes, to classify PE patients, the criteria of 2001 described by the International Society for the Study of Hypertension in Pregnancy were used. So, all of the PE patients (at least) had proteinuria.**

**We address this point in the discussion, since indeed there are new guidelines state that PE is diagnosed based on the presence of de novo hypertension after 20 weeks gestation accompanied by one of the following: proteinuria, acute kidney injury, liver dysfunction, neurological features, hemolysis or thrombocytopenia, or fetal growth restriction.**

Also, I think it would be beneficial to show the offspring parameters according to the certain HDP that the mother experienced.

**We added an extra table with the requested information: offspring parameters according to the certain type of HDP (S1 Table).**

There are a couple of grammatical changes that I would also suggest. A paper reads best when it is written with a consistent point of view. So instead of saying that "we found that the presence of maternal HDP was inversely associated with offspring triglyceride levels...", it may be better to reword this type of sentence to " the presence of maternal HDP was found to be inversely associated with offspring triglyceride levels..." to keep the entire paper written from the third person point of view.

**We thank the reviewer for this important point, and we made changes as suggested.**

Additionally, the in text references should be listed before the period.

**We made adjustments accordingly, and therefore listed references before the period.**

There were also a couple of times were the data was not provided. Do you have this data to make available? If not, I wouldn't include it in the results section.

**Now, that data is provided within the manuscript.**

Overall, this study provides some good information, but would definitely be strengthened if the offspring were continually monitored through adulthood, as you mentioned in the discussion.

**Indeed, we agree with the reviewer this is one of the most important discussion points within this manuscript.**

Reviewer #2: The article conducted a systematic review to evaluate the relationship between maternal hypertensive disorder during pregnancy and the offspring cardiometabolic risk. The paper observed a negative association between maternal hypertension disorder and offspring BMI at 12 month. However, some major questions are also noticed:
The symptoms of cardiometabolic disorder include high blood sugar, high blood pressure, high triglycerides, low HDL, belly fat/central adiposity. One of the parameters used to determine cardiometabolic disorder in the paper is fat mass index, which will also include peripheral. Why didn’t the author use the waist to hip ratio or waist circumference?

**We agree with the reviewer this measure is a good outcome measurement for cardiometabolic health. However, these measures may be imprecise and do not give any insight into the amount or differential effects of visceral and subcutaneous fat compartments.**

HELLP is one of the pregnancy disorders the author evaluated in the following studies, which is diagnosed by AST, ALT, etc. However, HELLP is not always accompanied by high blood pressure. Did the author determine that the patient with HELLP indeed had hypertension?

**Indeed, the author is right that HELLP does not always have to be accompanied by hypertension or proteinuria. Since the HELLP syndrome is one serious manifestation of pre-eclampsia we choose to take this disorder into account as well, despite the possibility of the absence of hypertension. Lastly, gestational hypertension and pre-eclampsia: HDP cases included 293 cases of GH, 139 cases of PE, 14 cases of HELLP, 20 cases of PE and HELLP and 25 cases of superponated PE/HELLP. Meaning, that holding onto the hypertension criterion, only 14 cases would be taken out of the analyses (since 20 had PE and HELLP, and 25 cases had superponated PE/HELLP and thus already manifested hypertension in pregnancy.)**

The author found that maternal HDP is negatively associated with offspring BMI, but not ponderal index, while the author mentioned that PI could be a better measurement for infancy. It may be necessary to include the data of PI and maternal HDP.

**We did not observe differences in results when we used the ponderal index as outcome measurement instead of BMI at 2, 6, 12 and 36 month. These results are now included in Table 2.**

There is a common body mass measurement for infants under 2 years old, BMI for age according to WHO. This may give a more consistent result.
**We thank the reviewer for this point. We are aware that both ponderal index and BMI for age are strongly correlated. Additionally, changes in PI/BMI in early infancy (until 10 years of age) are associated with greater fat-mass in later life. Moreover, we found comparable results in the analyses with both offspring BMI and the measure which is more frequently used in a clinical setting: ponderal index.**

In addition, there is a minor issue: In the method section, about pregnancy dating, the author mentioned CRL measurement. However no full name was given in the previous content.

**Indeed, we apologize for this, and now added the full name into the manuscript, which is crown-rump length.**

**S1 Table. Offspring parameters according to the certain HDP that the mother experienced.**

| **Study population**  **n = 7794** | **Gestational hypertension (n=293)** | **PE**  **(n=139)** | **HELLP**  **(n=14)** | **PE and HELLP**  **(n=20)** | **Superponated PE/HELLP (n=25)** |
| --- | --- | --- | --- | --- | --- |
| **Child characteristics** |  |  |  |  |  |
| Male sex, n (%) | 139 (47.4%) | 68 (48.9%) | 8 (57.1%) | 11 (55.0%) | 13 (52.0%) |
| Gestational age at birth  (weeks) | 40.1 (37.0-42.1) | 40.0 (37.0-41.9) | 40.1 (38.0-42.2) | 40.5 (37.8-41.4) | 40.4 (36.1-41.9) |
| Birth weight (grams) | 3407 (528) | 3353 (553) | 3616 (504) | 3360 (380) | 3493 (522) |

Abbreviations: BMI, body mass index; HDP, hypertensive disorder of pregnancy. Values are percentages for categorical variables, means (SD) for continuous variables with a normal distribution, or medians (5^th^, 95^th^ percentile) for continuous variables with a skewed distribution.

|  | **Cardiometabolic**  **risk factor** |  |  |  |  |
| --- | --- | --- | --- | --- | --- |
|  |  | **β (95% CI)** | **p-value** | **Standard error** | **R^2^** |
| **2 months** | **BMI** | -0.05 (-0.18 ; 0.09) | 0.50 | 0.07 | 0.03 |
|  | **PI** | -0.08 (-0.24 ; 0.07) | 0.31 | 0.08 | 0.04 |
| **6 months** | **BMI** | -0.09 (-0.21 ; 0.04) | 0.17 | 0.06 | 0.04 |
|  | **PI** | -0.11 (-0.23 ; 0.02) | 0.09 | 0.006 | 0.04 |
| **12 months** | **BMI** | **-0.15 (-0.27 ; -0.03)** | **0.02** | 0.06 | 0.02 |
|  | **PI** | **-0.17 (-0.29 ; -0.05)** | **0.01** | 0.06 | 0.01 |
| **36 months** | **BMI** | -0.002 (-0.13 ; 0.13) | 0.97 | 0.07 | 0.05 |
|  | **PI** | -0.06 (-0.19 ; 0.07) | 0.35 | 0.07 | 0.04 |
| **6 years** | **BMI**¶ | 0.03 (-0.07 ; 0.13) | 0.53 | 0.05 | 0.11 |
|  | **SBP** | 0.05 (-0.07 ; 0.16) | 0.42 | 0.06 | 0.03 |
|  | **DBP** | 0.10 (-0.01 ; 0.21) | 0.09 | 0.06 | 0.03 |
|  | **Fat mass index** ¶ | 0.04 (-0.06 ; 0.14) | 0.42 | 0.05 | 0.23 |
|  | **Cholesterol**¶ | 0.01 (-0.12 ; 0.14) | 0.87 | 0.07 | 0.02 |
|  | **Triglycerides**¶ | -0.10 (-0.23 ; 0.03) | 0.14 | 0.07 | 0.02 |
|  | **Pulse** | 1.18 (-0.01 ; 2.37) | 0.05 | 0.61 | 0.04 |
|  | **Cardiometabolic risk factor clustering** | 1.16 (0.84 ; 1.60) | 0.38 | 0.04 | 0.02 |
