# Supplementary material for: Maternal hypertensive disorders in pregnancy and early childhood cardiometabolic risk factors: The Generation R Study
Source: PLoS One. 2021 Dec 23;16(12):e0261351. doi: 10.1371/journal.pone.0261351 (PMC8699579; doi:10.1371/journal.pone.0261351)
Supplement: Attachment — Submitted filename: Response to Reviewers PONE-D-21-12261R1.docx [file pone.0261351.s003.docx]

**We thank the reviewers and the editorial office of PLOS ONE for the opportunity to again submit major revisions. Our answers (in bold) are as follows**

Reviewers' comments:
Reviewer's Responses to Questions

Comments to the Author

1. If the authors have adequately addressed your comments raised in a previous round of review and you feel that this manuscript is now acceptable for publication, you may indicate that here to bypass the “Comments to the Author” section, enter your conflict of interest statement in the “Confidential to Editor” section, and submit your "Accept" recommendation.

Reviewer #1: All comments have been addressed

Reviewer #2: All comments have been addressed

2. Is the manuscript technically sound, and do the data support the conclusions?
The manuscript must describe a technically sound piece of scientific research with data that supports the conclusions. Experiments must have been conducted rigorously, with appropriate controls, replication, and sample sizes. The conclusions must be drawn appropriately based on the data presented.

Reviewer #1: Yes

Reviewer #2: Yes

3. Has the statistical analysis been performed appropriately and rigorously?

Reviewer #1: Yes

Reviewer #2: N/A

4. Have the authors made all data underlying the findings in their manuscript fully available?

The [PLOS Data policy](http://www.plosone.org/static/policies.action#sharing) requires authors to make all data underlying the findings described in their manuscript fully available without restriction, with rare exception (please refer to the Data Availability Statement in the manuscript PDF file). The data should be provided as part of the manuscript or its supporting information, or deposited to a public repository. For example, in addition to summary statistics, the data points behind means, medians and variance measures should be available. If there are restrictions on publicly sharing data—e.g. participant privacy or use of data from a third party—those must be specified.

Reviewer #1: Yes

Reviewer #2: Yes

5. Is the manuscript presented in an intelligible fashion and written in standard English?

PLOS ONE does not copyedit accepted manuscripts, so the language in submitted articles must be clear, correct, and unambiguous. Any typographical or grammatical errors should be corrected at revision, so please note any specific errors here.

Reviewer #1: Yes

Reviewer #2: Yes

6. Review Comments to the Author

Please use the space provided to explain your answers to the questions above. You may also include additional comments for the author, including concerns about dual publication, research ethics, or publication ethics. (Please upload your review as an attachment if it exceeds 20,000 characters)

Reviewer #1:

I found very few instances of grammatical errors, so one more round of proof reading should occur before submission.

**Apologies, the manuscript was thoroughly checked again. Some mistakes have crept in after formatting the manuscript from ‘track changes’ to ‘no track changes’.**

Additionally, in Table 1, which defines the maternal characteristics of the sample populations, the systolic and diastolic blood pressures are only slightly elevated. 118 vs 119 (systolic) and 69.0 vs 69.8 (diastolic) between the women without HDP vs those with HDP respectively. As mentioned in the methods, the defining characteristics of two of the three pregnancy complications are defined as systolic/diastolic blood pressures of greater than 140/80, which is relatively higher than the mean for the mothers with HDPs. Can the authors explain why the mean of the HDP was so low? Is it due to the HELLP syndrome mothers not exhibiting high blood pressure?

**Thank you for addressing this important point. There are 3 possible explanations for the relative low (mean) blood pressures and small differences in the blood pressures between the two groups.**

1. **Timing of measurement.**

**The maternal blood pressure was measured in the third measurement period of the study, i.e. ‘late pregnancy’, which was defined as ≥25 weeks gestational age. However, hypertensive disorders of pregnancy (such as preeclampsia) are most prevalent at 34 or more weeks of gestation,** **with an incidence of 2.7%.** *von Dadelszen et al. Subclassification of preeclampsia. Hypertens Pregnancy. 2003;22(2).*

*Lisonkova et al. Incidence of preeclampsia: risk factors and outcomes associated with early- versus late-onset disease. Am J Obstet Gynecol. 2013;209(6).* **We retrieved the diagnosis of a hypertensive disorder of pregnancy in retrospect from medical records. These blood pressure measurements therefore do not reflect the blood pressure measurement at the time of diagnosis.**

2. **Missing data**

**Before they were able to attend the measurement at the Generation R study Centre, women could already be hospitalized due to a hypertensive disorder of pregnancy.**

**So, these women are classified as having a HDP, and we have follow up data with regard to their infant cardiometabolic health. However, we then lack the data of (high) blood pressure in the latest phase of pregnancy.**

3. **Heterogeneity of the hypertensive disorders.**

**Indeed, hypertension isn’t one of the criteria to diagnose ‘HELLP syndrome’, thereby the women with this diagnosis do not affect the mean blood pressure in the ‘HDP-group’.**

**Additionally, it is interesting to mention that the maximum blood pressures are 185 (systolic) and 118 (diastolic), indicating that indeed there are women with a hypertensive disorder of pregnancy in this group already.**

**These points were added to the manuscript, chapter ‘Results’ lines 8-18:**

***Differences in systolic and diastolic blood pressure between the groups of women with and without a HDP were small. Moreover, mean blood pressures in the HDP group were relatively low. This could be explained by the fact that maternal blood pressure was measured in late pregnancy, i.e. ≥25 weeks of gestation. Thereby, the onset of a HDP could be (long) after the blood pressure measurement at the Generation R study research facility. Then, the blood pressure measurement in Table 1 does not reflect blood pressure at the time of diagnosis. Second, a woman may be hospitalized due to a HDP, before she could attend the Generation R research facility: then her blood pressure measurement was missing. Lastly, the relatively low blood pressure could be due to the heterogeneity of the HDP group. Since hypertension isn’t one of the criteria to diagnose ‘HELLP syndrome’, the women with HELLP in the HDP group do not increase the mean systolic or diastolic blood pressure.***

Reviewer #2: (No Response)

7. PLOS authors have the option to publish the peer review history of their article ([what does this mean?](https://journals.plos.org/plosone/s/editorial-and-peer-review-process#loc-peer-review-history)). If published, this will include your full peer review and any attached files.
If you choose “no”, your identity will remain anonymous but your review may still be made public.
Do you want your identity to be public for this peer review? For information about this choice, including consent withdrawal, please see our [Privacy Policy](https://www.plos.org/privacy-policy).

Reviewer #1: No

Reviewer #2: No

[NOTE: If reviewer comments were submitted as an attachment file, they will be attached to this email and accessible via the submission site. Please log into your account, locate the manuscript record, and check for the action link "View Attachments". If this link does not appear, there are no attachment files.]

While revising your submission, please upload your figure files to the Preflight Analysis and Conversion Engine (PACE) digital diagnostic tool, <https://pacev2.apexcovantage.com/>. PACE helps ensure that figures meet PLOS requirements. To use PACE, you must first register as a user. Registration is free. Then, login and navigate to the UPLOAD tab, where you will find detailed instructions on how to use the tool. If you encounter any issues or have any questions when using PACE, please email PLOS at [figures@plos.org](mailto:figures@plos.org). Please note that Supporting Information files do not need this step.

**PACE corrected files were uploaded.**
